# SECs (Sinusoidal Endothelial Cells), Liver Microenvironment, and Fibrosis

**DOI:** 10.1155/2017/4097205

**Published:** 2017-02-15

**Authors:** Vaishaali Natarajan, Edward N. Harris, Srivatsan Kidambi

**Affiliations:** ^1^Department of Chemical and Biomolecular Engineering, University of Nebraska, Lincoln, NE, USA; ^2^Department of Biochemistry, University of Nebraska, Lincoln, NE, USA; ^3^Nebraska Center for Integrated Biomolecular Communication, University of Nebraska, Lincoln, NE, USA; ^4^Fred & Pamela Buffett Cancer Center, University of Nebraska Medical Center, Omaha, NE, USA; ^5^Nebraska Center for the Prevention of Obesity Diseases, University of Nebraska, Lincoln, NE, USA; ^6^Nebraska Center for Materials and Nanoscience, University of Nebraska, Lincoln, NE, USA; ^7^Mary and Dick Holland Regenerative Medicine Program, University of Nebraska Medical Center, Omaha, NE, USA

## Abstract

Liver fibrosis is a wound-healing response to chronic liver injury such as alcoholic/nonalcoholic fatty liver disease and viral hepatitis with no FDA-approved treatments. Liver fibrosis results in a continual accumulation of extracellular matrix (ECM) proteins and paves the way for replacement of parenchyma with nonfunctional scar tissue. The fibrotic condition results in drastic changes in the local mechanical, chemical, and biological microenvironment of the tissue. Liver parenchyma is supported by an efficient network of vasculature lined by liver sinusoidal endothelial cells (LSECs). These nonparenchymal cells are highly specialized resident endothelial cell type with characteristic morphological and functional features. Alterations in LSECs phenotype including lack of LSEC fenestration, capillarization, and formation of an organized basement membrane have been shown to precede fibrosis and promote hepatic stellate cell activation. Here, we review the interplay of LSECs with the dynamic changes in the fibrotic liver microenvironment such as matrix rigidity, altered ECM protein profile, and cell-cell interactions to provide insight into the pivotal changes in LSEC physiology and the extent to which it mediates the progression of liver fibrosis. Establishing the molecular aspects of LSECs in the light of fibrotic microenvironment is valuable towards development of novel therapeutic and diagnostic targets of liver fibrosis.

## 1. Introduction

Liver Fibrosis is one of the leading causes of liver-related morbidity and mortality in the United States with an estimated health care cost of $103 billion per year ($1,613 per patient). Hepatic fibrosis occurs in response to chronic liver injury initiated by several factors including alcoholic/nonalcoholic fatty liver disease and viral hepatitis [1–4]. Fibrosis is a chronic condition which initiates a cascade of biochemical and biophysical changes in the liver microenvironment causing necrosis and apoptosis of hepatocytes (highly specialized epithelial cells) and liver sinusoidal endothelial cells (LSECs), through the release of inflammatory mediators and profibrotic cytokines and activation of hepatic stellate cells. Further exacerbation of this chronic wound-healing response results in excess deposition and decreased turnover of extracellular matrix (ECM) proteins (e.g., collagen). The increased density of ECM results in increased matrix stiffness, and recent studies demonstrate that this phenomenon correlates and contributes to the progression of liver fibrosis [5–8]. Recent clinical reports and animal studies have revealed that fibrosis could be reversible [9–13]. While fibrosis is reversible in its initial stages, uninterrupted fibrosis may lead to cirrhosis. The exact point when fibrosis becomes irreversible requires further investigation. Despite significant advances in understanding hepatic fibrosis and defining targets for therapy, there are no antifibrotic drugs yet approved for clinical use in patients with advanced liver disease. The parenchymal and nonparenchymal cells in the liver microenvironment play a unique role in the pathology of liver fibrosis. The progression and regression of liver fibrosis rely on a complex interplay between the hepatocytes, LSECs, stellate cells, and Kupffer cells and the noncellular components of the fibrotic microenvironment [14–20]. Understanding the cellular and molecular mechanisms involved in fibrosis progression has a number of clinical implications, including the development of therapeutic interventions to impede or reverse hepatic fibrosis, some of which are already available [1, 2, 21].

The high metabolic activity of the liver demands an efficient vasculature in the organ. The intricate network of capillaries of the liver is lined by liver sinusoidal endothelial cells (LSECs), a specialized endothelial cell type that is phenotypically different from vascular endothelial cells [22]. LSECs are characterized by their unique morphological characteristics such as lack of a basement membrane in the endothelium and presence of open fenestrations [22]. These fenestrations are clustered throughout the cytoplasm to form dynamic sieve plates and facilitate the steric transport of cargo from the lumen (sinusoidal space) to the space of Disse and into the parenchyma [23]. The alterations in the diameter and frequency of fenestrations on LSECs are correlated with several liver injuries, toxins, and diseases and have implications in the loss of overall liver function [23, 24]. LSECs also possess a characteristically high scavenger function for a diverse array of macromolecules. These cells contain a large number of endocytic vesicles that perform degradation and recycling of macromolecular wastes from lumen such as extracellular matrix breakdown, immune complexes, and lysosomal enzymes [25]. Additionally, LSECs play an active role in the immune regulation of the organ through bacterial processing, leukocyte adhesion, and viral clearance [26–28]. These cells also maintain the hemodynamics of liver capillaries by actively responding to the varying intrahepatic blood flow and pressure [29]. Additionally, the paracrine signaling between LSECs and hepatocytes is crucial for the functional maintenance of the parenchyma. Similar to the functional importance of LSECs in a healthy liver, these cells are recognized as early regulators in the progression of liver fibrosis.

**Figure 1:**
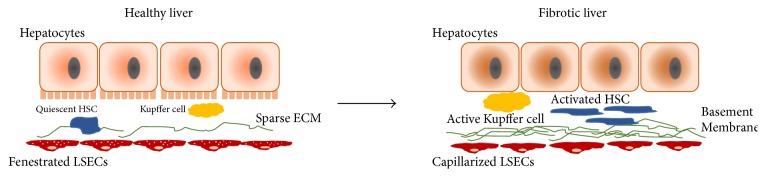
Morphological changes in LSECs during liver fibrosis. Hepatocytes and liver sinusoidal endothelial cells are separated by the space of Disse that contains minimal extracellular matrix (ECM) in a healthy liver. Quiescent hepatic stellate cells reside in the space of Disse. The Kupffer cells undergo phenotypic change to become more inflammatory. The fenestrations on LSECs allow for solute transport. In a fibrotic liver, the stellate cells are activated, a basement membrane is formed in the endothelium, and the LSECs are defenestrated.

Liver injuries leading to fibrosis result in a drastic alteration in the LSEC phenotype. The loss in fenestration and the appearance of basement membrane in the space of Disse are observed early in a fibrotic liver, a process referred to as capillarization. DeLeve and co-workers demonstrated that the capillarization of LSECs (loss in fenestrations) precedes the onset of fibrosis and acts as a gatekeeper event for the progression of liver fibrosis [30]. Several secretory changes such as loss of endothelial nitric oxide synthase (eNOS) activity and overexpression of endothelin-1 also accompany fibrosis and mediate portal hypertension in the liver [31]. Additionally, capillarized LSECs contribute to the ECM accumulation in the fibrotic liver in the form of collagen and fibronectin synthesis. Fibrosis is also influenced by a reversal of LSEC function from tolerogenic to proinflammatory and immunogenic; this phenomenon contributes to both heightened inflammatory milieu and altered intrahepatic immunity [32, 33]. Changes in LSECs during liver fibrosis result in a cascade of autocrine and paracrine responses and warrant a systematic analysis of the role of LSECs in the fibrotic microenvironment to further the research towards early detection and therapy for liver fibrosis (Figure 1). Therefore, it is crucial to understand the role of dynamic changes in liver microenvironment during liver fibrosis pertaining to the subtle but pivotal changes in LSEC physiology, and the extent to which it mediates the progression of liver fibrosis.

## 2. Mechanical Environment of the Fibrotic Liver and LSECs

An excessive accumulation of ECM proteins and remodeling is synonymous with liver fibrosis and results in a dramatic change in the mechanical microenvironment, particularly in the stiffness of the organ [34]. Increase in the liver stiffness is currently the most clinically relevant diagnostic marker, and several elastography-based techniques are utilized to correlate the severity of liver fibrosis with the organ stiffness value [35–37]. Recent studies have highlighted the profound impact of biophysical attributes of ECM, including dynamic changes in matrix stiffness, as a key mechanism of modulating hepatic stellate cell activation and one of the contributors to fibrosis disease progression [38–42]. Animal studies have shown that increase in liver stiffness precedes the development of fibrosis in an iterative carbon tetrachloride model of rat liver fibrosis [43]. Wells and co-workers have demonstrated that matrix stiffness regulates the myofibroblastic differentiation of two precursor populations, hepatic stellate cells and portal fibroblasts [40]. Hepatic stellate cells cultured on stiff environment (8–12 kPa) developed fibrosis-like features of myofibroblast phenotype, including enhanced cell spreading, *α*-smooth-muscle actin (*α*SMA) expression, and stress fiber organization. Recently, our group has demonstrated that physiological ranges of matrix stiffness regulated primary hepatocyte morphology. Hepatocytes cultured on soft (healthy) substrates displayed a more differentiated and functional phenotype for a longer duration as compared to stiff (fibrotic-like) substrates [44]. Matrix stiffness was demonstrated to alter hepatocytes function wherein hepatocytes on soft substrates retained 2.7-fold higher cytochrome P450 (CYP) activity on day 7 in culture, as compared to the control group on standard tissue culture surface. In addition, we observed that the epithelial cell phenotype was better maintained on soft substrates as indicated by higher expression of hepatocyte nuclear factor 4 alpha, cytokeratin 18, and connexin 32 [44]. Weaver and co-workers have demonstrated that fibrotic levels of matrix stiffness significantly inhibit hepatocyte-specific functions by inhibiting the HNF4*α* transcriptional network mediated through the Rho/Rho-associated protein kinase pathway [45]. These data suggest that early increase in liver stiffness is mechanistically important in regulating individual cellular responses to tissue injury. A majority of the current work studying the role of tissue stiffness in pathogenesis of hepatic fibrosis focuses on hepatic stellate cells. The effect of varying mechanical cues on LSECs is particularly interesting; however, this has not been extensively explored until recently. Since the capillarization of LSECs is also an initial trigger for fibrosis, establishing the correlation between the mechanical cues and LSECs is of prime importance towards achieving an early therapeutic intervention. This is an important question because the mechanistic cause of liver failure in cirrhosis is not fully understood and there is evidence that cells including hepatocytes and LSECs from a cirrhotic liver may regain lost function when exposed to a healthy liver microenvironment [46, 47]. Understanding the effect of increased matrix stiffness during the course of liver fibrosis on LSEC function will provide more insight into the role of matrix rigidity as a contributor to the disease progression.

Endothelial cell behavior is largely regulated by the mechanical cue of shear stress dictated by flow of blood through the lumen of blood vessels and capillaries [48]. In case of liver microvasculature, despite the low flow rate of blood, the narrow capillary diameter results in a significant shear stress generation [49, 50]. The fenestrations are especially sensitive to shear stress in the lumen. Studies with animal models have demonstrated that introduction of a high perfusion pressure through the portal vein resulted in the fusion and enlargement of the LSEC fenestrae, resulting in an abnormal transport of chylomicrons to the parenchyma [51]. Several studies have also attempted identification of changes in the molecular signature change that accompanies LSECs under abnormal shear stress. Employing an in vitro culture model of LSECs with a microfluidic setup recreated the shear stress of fluid flow and demonstrated that LSECs cope with increasing shear stress in the microenvironment by increasing nitric oxide (NO) synthesis [29]. In fibrotic livers, the microvasculature remodeling results in increased vascular resistance and consequent increase in shear stress [52]. Rodent models of liver fibrosis have demonstrated that LSECs had a significantly lower expression of NO and nitric oxide synthase (NOS) [53]. DeLeve and co-workers demonstrated that the NO signaling pathway regulates the maintenance of LSEC differentiated phenotype indicating that the autocrine production of NO by LSECs regulates the VEGF mediated pathway [54]. LSECs in cirrhotic livers with evident portal hypertension overexpress Kruppel-like factor 2 (KLF2) to cope with the abnormal hemodynamics of the liver [55]. In the liver, the transcription factor KLF2 is induced early during progression of cirrhosis to lessen the development of vascular dysfunction; nevertheless, its endogenous expression results are insufficient to attenuate establishment of portal hypertension and aggravation of cirrhosis [56, 57]. The important role of shear stress in endothelial dysfunction and the effect of restoring the flow rate on the LSEC function need further investigation. In an attempt to investigate the phenotype restoration of LSECs, Hwa et al. demonstrated that LSECs can be rendered functionally stable when maintained in perfusion cultures with laminar flow of media mimicking the shear stress condition of a healthy liver [58]. In another study, Domansky and co-workers retained the viability of LSECs for a prolonged duration by maintaining a physiological flow rate in a bioreactor [59]. LSECs cultured in perfused bioreactor retained expression of the functional marker sinusoidal endothelial 1 (SE-1) and exogenous supportive endothelial growth factors like vascular endothelial growth factor (VEGF) upto 13 days after seeding. Furthermore, the retention of the endothelial phenotype was observed to be dependent on the flow rate and the oxygen concentration in the perfused multiwell.

The mechanical microenvironment, especially in the form of tissue stiffness and stretching forces, is proven to activate hepatic stellate cells and promote liver fibrosis progression [40, 60]. Since the paracrine signaling between activated stellate cells and LSECs is known to be instrumental in the capillarization of LSECs, mechanical cues can also be considered to have an indirect effect on LSECs [54]. Juin and co-workers showed that increased ECM matrix rigidity increased the number of podosomes (actin-rich structures involved in motility and proteolysis) formed in LSECs suggesting that the cells responded to mechanical stress and underwent cytoskeletal remodeling [61]. Similarly, human LSECs lose the fenestrations and demonstrate increased stress fiber formation when subjected to high stiffness microenvironment [62]. Also, we have demonstrated that matrix stiffness regulates LSEC morphology and function eliciting cell behavior akin to those observed in animal models of liver fibrosis [63]. The understanding of the direct effects of various mechanical forces such as stiffness, compression, shear stress, and stretch on LSECs will contribute to elucidating the role of LSECs in liver fibrosis and identification of therapeutic targets in the mechanotransduction pathways to effect reversal of fibrosis.

## 3. Extracellular Matrix of Fibrotic Liver and LSECs

A healthy liver contains a significantly low amount of ECM as compared to some other organs, constituting approximately 3% of the liver area [64]. The most prominent macromolecules of the healthy liver ECM are collagens of types I, III, IV, and V, glycoproteins such as fibronectin, laminin, and tenascin, glycosaminoglycans such as heparin, chondroitin sulfate, and hyaluronic acid, and proteoglycans such as perlecan and decorin [65, 66]. Healthy LSECs also contribute to the ECM by producing a modest amount of macromolecules such as collagen type IV and fibronectin [67]. In the event of liver fibrosis, the quantity of ECM increases many fold but, interestingly, the ECM composition remains relatively unaltered.

In a healthy liver, the space of Disse has a low density of ECM proteins which enables easy transport of cargo from the blood vessel lumen to various liver cells thereby playing a crucial role in the functional maintenance of the cells [67]. The typical sparse basement membrane is made up of collagen type IV and laminin but during early stages of liver fibrosis, the space of Disse (perisinusoidal space) undergoes changes both in terms of the residing cells and the ECM composition. A network of fibrillar collagen combined with excess collagen type IV and laminin increases and forms a basement membrane, as witnessed in clinical studies and animal models [68–71]. Studies suggest that once a substantial basement membrane appears in the liver endothelium, the changes in LSEC phenotype and fenestrations become virtually irreversible [72].

Recent studies have established that LSECs can be an active contributor to the excessive ECM in the event of liver fibrosis [18, 20, 73, 74]. Identification of the specific ECM expressed by LSECs can be challenging since their scavenging action of ECM fragments present in the lumen results in ambiguity regarding the cellular source of the protein. Several animal and clinical studies have identified the specific contributions of LSECs towards the fibrotic ECM. Maher and McGuire demonstrated that, after liver injury, LSECs displayed an increase in the mRNA levels of collagen type I [75]. Similarly, rodent studies by Neubauer et al. showed that injured LSECs synthesized a higher amount of collagen type IV [76]. Animal studies have also displayed that LSECs can synthesize fibronectin, a necessary structural unit of the liver ECM [77]. Apart from the typical structural/cell adhesion role that is attributed to cellular fibronectin, LSECs demonstrate a higher expression of the EIIIA fragment of fibronectin which can play the role of an active bioligand triggering the wound-healing response in the organ [78].

The excessive accumulation and the altered structural aspects of ECM in a fibrotic liver affect the phenotype of LSECs, directly and indirectly. The direct effect of the excessive collagen type I was explored by McGuire et al. where they showed that presence of interstitial collagen fibers resulted in the defenestration of LSECs [79]. Apart from the physical barrier due to the basement membrane, the defenestration of LSECs further compromises the ability for the exchange of molecules between parenchyma and the lumen. Similarly, Shakado et al. demonstrated that an increase in laminin concentration of the culture substrate resulted in a loss in endothelial pores [80]. Another interesting study by Sellaro et al. showed that LSECs cultured on decellularized liver ECM maintained a fenestrated phenotype for a prolonged period when compared with decellularized ECM of other organs [81].

The altered ECM during the progression of liver fibrosis also results in changes in the profile of cell-matrix adhesion molecules expressed by LSECs. Couvelard and co-workers demonstrated that LSECs overexpress several integrins that act as laminin receptors, including *α*_6_*β*_1_ and attributed this to the increase in laminin in the space of Disse [82]. These studies suggest the responsiveness of LSECs to the various components of the ECM. With respect to indirect effects, ECM acts as means for the attachment and storage of several cytokines and growth factors that when activated can result in changes in the LSEC behavior [83]. A variety of growth factors such as TGF-*β*, FGFs, TNF-*α*, and PDGF are covalently or noncovalently bound to the ECM molecules of collagens and fibronectin [84].

## 4. Cross-Talk between LSECs and the Liver Parenchyma (Hepatocytes)

Communication between parenchyma of the liver and LSECs is crucial in the healthy liver as well as in the progression of liver fibrosis. Structural features of LSECs such as fenestrations ensure regulated bidirectional transport of metabolites and solutes between blood and parenchyma. Loss of fenestration has implications in the functional loss in hepatocytes and development of secondary metabolic diseases such as atherosclerosis. Altered fenestration dimensions result in irregular transport of chylomicrons and access to hepatocytes thereby altering the lipid metabolism, and higher release of cholesterol by these cells [85].

The signaling pathway underlying the cross-talk between hepatocytes and LSECs has recently received significant attention. LSECs regulate the functional maintenance and regeneration of hepatocytes through paracrine action of angiogenic trophogens such as hepatic growth factors (HGF) and Wnt2; LSECs regulate the functional maintenance and regeneration of hepatocytes [86]. This phenomenon was further corroborated by Yamane et al., wherein the paracrine nature of LSEC and hepatocyte communication mediated by VEGF-Flt receptor family on LSECs and HGF-Met receptor groups on hepatocytes were validated [87].

Recent studies have garnered attention to exosomes as a potential means of transfer of macromolecules between different cell types. Exosomes are vesicular structures of less than 100 nm diameter that are taken up by cells in a nonreceptor mediated manner. Microparticles released by LSECs in cirrhotic patients were shown to contain signaling molecules that regulate hepatocyte behavior [88]. Another study demonstrates that hepatocytes subjected to lipotoxicity released exosomes that are rich in VNN1 and the presentation of VNN1 on the exosome surface results in uptake by endothelial cells making them proangiogenic in phenotype [89].

## 5. Cross-Talk between LSECs and the Liver Nonparenchymal Cells

One of the primary pathophysiological responses to LSEC injury is the early activation of hepatic stellate cells (HSCs) that constitute the most fibrogenic cellular species of the liver. These activated HSCs exhibit the phenotypic traits of higher contractility and proliferation, secrete high amounts of collagen type I and TGF-*β*, possess a higher expression of *α*-SMA, and lose the retinoid storage function [90]. Multiple cytokines have been implicated in triggering the activation of HSCs and several of them are released by injured LSECs. Capillarized LSECs secrete fibronectin EIIIA which activate HSCs [78]. Similarly, a decrease in the KLF-2 factor in LSECs results in HSC activation and in vitro studies show that overexpressing KLF-2, on the other hand, can restore the quiescent phenotype of HSCs [57]. The multifaceted role of TGF-*β* as a key mediator of fibrosis is well established [60]. LSECs play a crucial role in activation of TGF-*β*1 through plasmin which in turn activates HSCs [91]. Similarly, PDGF also mediates the activation of HSCs in a paracrine (LSECs secrete PDGF) and autocrine manner.

Communication between LSECs and HSCs is vital for the functional maintenance of LSECs as well [91]. Similar to hepatocytes, quiescent HSCs also have been reported to secrete VEGF that plays a crucial role in the maintenance of fenestrations in LSECs in both a NO-dependent and NO-independent manner [92]. Activated HSCs were shown to release microparticles loaded with Hedgehog signaling molecules that interact with LSECs and alter their gene expression profile [93]. Similarly, exosomes from endothelial cells that contained sphingosine kinase 1 (SK1) and demonstrated fibronectin expression were shown to regulate the activation of HSCs [94].

Kupffer cells are the macrophages of the liver and play a crucial role scavenging foreign materials that end up in the portal circulation [95]. They are also responsible for the initiation of inflammation by releasing active cytokines and reactive oxygen species as a response to hepatotoxin mediated injury to hepatocytes or biliary epithelial cells. In a fibrotic liver, Kupffer cells overexpress platelet derived growth factor (PDGF) which acts as the primary mitogen for activated stellate cells, hence indirectly driving forth fibrogenesis [96, 97]. In the specific case of alcohol mediated injury, the Kupffer cells get activated and produce TNF-*α* that evokes parenchymal stress response [98]. Despite advances in isolating their role in liver disease progression, the cross-talk between Kupffer cells and LSECs remains largely unexplored. In a rodent model with liver injury due to sepsis, Hutchins et al. demonstrated that the interaction between PD-1 expressing Kupffer cells and PD-L1 expressing LSECs resulted in endothelial dysfunction [99]. Similarly, Arii and Imamura demonstrated that blocking the signaling from Kupffer cells in cold-preserved liver resulted in restoration of LSEC phenotype. The study also demonstrates that an increase in ICAM-1 expression on LSECs is regulated by secretion of TNF-*α* by Kupffer cells [100]. In an attempt to establish the communication between Kupffer cells and LSECs, an in vitro study by Ford et al. demonstrated that in a fibrotic model, cross-talk between LSECs and Kupffer cells resulted in a loss of fenestrations and increase in CD31 expression [62].

## 6. Therapeutic Interventions Targeting LSECs and Vasculature for Liver Disease

Restoration of LSEC phenotype could present a potential route for promoting the regression of fibrosis. Recent studies further our understanding of the role of LSECs in directly mediating fibrosis resolution. Decreasing the excessive collagen in a fibrotic liver through MMPs and TIMPs has been of interest for a while now but a study by Malovic et al. demonstrated that LSECs possess mannose receptors that rapidly endocytose denatured collagen *α* chains from blood [101]. Enhancing the LSEC mediated removal of collagen fragments could be a potential target for therapeutic purposes. The role of VEGF has been controversial due to the conflicting nature of several studies. In an effort to hinder angiogenesis in the fibrotic livers, Fernandez et al. have demonstrated the importance of inhibiting the signaling pathways of VEGF and PDGF in order to decrease the expression of CD31 and VEGFR-2 in the endothelium [102]. On the other hand, fibrosis resolution and maintenance of fenestration in LSECs have been achieved in rodent models through VEGF mediated pathway [103, 104]. Nitric oxide synthase (NOS) synthesized by LSECs is an important target for fibrosis resolution owing to the regulatory role it plays in portal hemodynamics. Decreased levels of NO are due to downregulation in the expression of eNOS in LSECs subjected to capillarization and the restoration of eNOS could have therapeutic implications [105]. Dill et al. showed that disruption of Notch1 signaling results in vascular remodeling of the liver and the intact signaling ensures the highly differentiated phenotype of LSECs is maintained, thus indicating the potential as an antiangiogenesis target for liver fibrosis [106].

Several experimental drugs have surfaced over the last decade that could potentially treat fibrosis by targeting the vascular function of the organ. Treatment of cirrhotic rats with tetrahydrobiopterin, an essential cofactor for the synthesis of NO, demonstrates a marked improvement in portal hypertension [107]. Another study combating the oxidative stress of the fibrotic liver demonstrated that administration of ascorbic acid in cirrhotic patients resulted in an increased bioavailability of NO, thereby resulting in a moderate alleviation of endothelial dysfunction [108]. Increasing superoxide population in the fibrotic livers of rodent models was treated with Tempol, a small superoxide dismutase (SOD) mimic, which resulted in the restoration of normal portal pressure [109]. In another multitargeted approach, Sunitinib administration to cirrhotic rats resulted in a decrease in hepatic vascular density and portal pressure [110]. Antifibrosis applications of statins in a study by Marrone et al. showed that statins can directly target LSECs in a fibrotic liver by triggering an upregulation of KLF2 expression, where KLF2 acts as a vasoprotective molecule of the liver endothelium [57].

## 7. Conclusions

Unchecked fibrosis of the liver results in a continual accumulation of ECM proteins and paves the way for replacement of parenchyma with nonfunctional scar tissue. The persistence of fibrotic state in the liver determines the severity of numerous challenges with respect to achieving complete restoration of liver health and some of these are reversal of vascular remodeling, excessive crosslinking between the collagen fibers, and loss in parenchyma [111]. Since phenotypic alteration in LSECs phenotype is one of the earliest events of fibrogenesis, investigating the molecular aspect of LSECs in the context of liver fibrosis progression will prove to be valuable towards early detection and a consequent early intervention of liver fibrosis.
